# A Technical Note on the PainChek™ System: A Web Portal and Mobile Medical Device for Assessing Pain in People With Dementia

**DOI:** 10.3389/fnagi.2018.00117

**Published:** 2018-06-12

**Authors:** Mustafa Atee, Kreshnik Hoti, Jeffery D. Hughes

**Affiliations:** ^1^School of Pharmacy and Biomedical Sciences, Faculty of Health Sciences, Curtin University, Bentley, WA, Australia; ^2^Division of Pharmacy, Faculty of Medicine, University of Pristina, Prishtina, Kosovo, Albania

**Keywords:** PainChek™, pain assessment system, artificial intelligence, automated facial recognition, dementia, smart device application, technology

## Abstract

**Background:** Pain in dementia is predominant particularly in the advanced stages or in those who are unable to verbalize. Uncontrolled pain alters the course of behaviors in patients with dementia making them perturbed, unsettled, and devitalized. Current measures of assessing pain in this population group are inadequate and underutilized in clinical practice because they lack systematic evaluation and innovative design.

**Objective:** To describe a novel method and system of pain assessment using a combination of technologies: automated facial recognition and analysis (AFRA), smart computing, affective computing, and cloud computing (Internet of Things) for people with advanced dementia.

**Methods and Results:** Cognification and affective computing were used to conceptualize the system. A computerized clinical system was developed to address the challenging problem of identifying pain in non-verbal patients with dementia. The system is composed of a smart device enabled app (App) linked to a web admin portal (WAP). The App “PainChek™” uses AFRA to identify facial action units indicative of pain presence, and user-fed clinical information to calculate a pain intensity score. The App has various functionalities including: pain assessment, pain monitoring, patient profiling, and data synchronization (into the WAP). The WAP serves as a database that collects the data obtained through the App in the clinical setting. These technologies can assist in addressing the various characteristics of pain (e.g., subjectivity, multidimensionality, and dynamicity). With over 750 paired assessments conducted, the App has been validated in two clinical studies (*n* = 74, age: 60–98 y), which showed sound psychometric properties: excellent concurrent validity (*r* = 0.882–0.911), interrater reliability (Kw = 0.74–0.86), internal consistency (α = 0.925–0.950), and excellent test-retest reliability (ICC = 0.904), while it possesses good predictive validity and discriminant validity. Clinimetric data revealed high accuracy (95.0%), sensitivity (96.1%), and specificity (91.4%) as well as excellent clinical utility (0.95).

**Conclusions:** PainChek™ is a comprehensive and evidence-based pain management system. This novel approach has the potential to transform pain assessment in people who are unable to verbalize because it can be used by clinicians and carers in everyday clinical practice.

## Introduction

In 2017, there are an estimated 962 million people aged 60 or over in the world, comprising 13% of the global population (United Nations Department of Economic Social Affairs Population Division, 2017). This number is projected to increase to 1.4 billion in 2030 and 2.1 billion in 2050, and could rise to 3.1 billion in 2100 (United Nations Department of Economic Social Affairs Population Division, 2017). The incidence of dementia doubles beyond the age of 65 and becomes prevalent by up to 50% over the age of 85 (Duthey, 2013). Currently, there are 50 million people living with the condition worldwide (ADI, 2017; WHO, 2017). Dementia is a clinical neurodegenerative syndrome characterized by progressively impaired cognition, communication (including pain self-reporting), and comprehension as well as the lack of ability to execute simple daily activities (Mitchell et al., 2009; WHO, 2017). Pain is common (up to 80%) in people with dementia but it often goes undetected and untreated, particularly in those who cannot verbalize or express their needs (Hadjistavropoulos et al., 2014). Uncontrolled pain alters the course of behaviors in patients with dementia making them perturbed, unsettled, and devitalized (Hadjistavropoulos et al., 2014).

Despite the availability of more than 35 observational-behavioral pain assessment tools for adults with communication difficulties (Hadjistavropoulos et al., 2014), including those with advanced dementia, none are currently approved by any regulatory agency, such as the United States' Food and Drug Administration (FDA), Australia's Therapeutic Goods Administration (TGA), and European Conformity (CE) mark. As of July 2017, the electronic Pain Assessment Tool (ePAT) [now known as PainChek™] received regulatory clearance as a Class 1 medical device for pain assessment and monitoring in adults who cannot verbalize (e.g., those with dementia) from the TGA and CE marking (ARTG, 2017). The mobile application (App) as a medical device has also been approved for use in other non-verbal adult populations such as those with other neurodegenerative disorders, intellectual disability, traumatic brain injury, aphasia, those receiving palliative care, and post stroke patients (ARTG, 2017). Further, none of the existing tools has their own electronic database, which collects data in real time. This is an important feature of clinical tools because it identifies the need for therapeutic intervention(s) in a timely fashion, which if successful lead(s) to improvement in patient outcomes. To achieve this goal we have developed an online secure portal linked to the tool App that can be accessed through various computing devices (e.g., PC, smart tablet, smartphone).

Current pain assessment tools in dementia also lack the innovative design and advanced technological characteristics. In a large meta-review of pain assessment tools in dementia Lichtner et al. (2014), argue the need for new tools that contain innovative characteristics to be able to transform the process of pain assessment in non-verbal older adults with dementia.

This note aims to describe a novel system called PainChek™ focusing on its conceptual foundation, clinical and technical contents, clinical use, and practical tips for use in clinical settings.

## Methods and results

### Conceptual foundation of painchek™ system (Figure 1)

In designing the PainChek™ system, the following conceptualizations were considered:

1. The subjective nature of pain i.e. individualized experience of pain as per its definition by the International Association for the Study of Pain (IASP) (Merskey and Bogduk, 1994).
2. The multi-dimensionality, complexity, and dynamicity of pain as a construct (Merskey and Bogduk, 1994).
3. The American Geriatric Society (AGS) Indicators of Persistent Pain were selected as a basic framework to enrich comprehensiveness and to meet the objectives of the tool (AGS Panel on Persistent Pain in Older Persons, 2002).
4. The temporality of pain and related behaviors, so that trends and patterns of pain scores provide a comprehensive clinical picture of the patient under assessment.
5. Objective description of key pain behaviors, such as Facial Action Coding System (FACS)—pain relevant expressions (Ekman et al., 1978).
6. Items sensitive to the presence and intensity of pain were selected on the basis of current evidence (clinical guidelines, previous studies, and other pain assessment tools in dementia).
7. Simple scoring mechanism. For clinicians and carers, it is difficult and highly subjective to make a distinction between whether a patient has mild, moderate or severe pain-related behaviors (Flaherty, 1996). We adopted binary scoring in the PainChek™ pain scale, because such mathematical basis is more predictive of event outcomes and less prone to error than ordinal rating (Ridley, 2002). These criteria are also linked to improved accuracy (Ridley, 2002).
8. Innovative technologies were considered in developing the system. Cognification and affective computing were conceptualized as a model in designing the App to provide a synergistic effect on the use of the tool (Kelly, 2016). Cognification integrates artificial (emotional) intelligence (AI) or affective computing e.g., automated facial recognition and analysis (AFRA), smart computing, and “Internet of Things” (IoT). Automation was integrated because the FACS requires lengthy training, and a certified skilled observer (coder), which render its use in clinical settings impractical (Craig et al., 2011). Smart device technology was selected because they are mobile, miniaturized, cost-efficient, easy to use, have high processing power, and they allow interoperability. IoT and cloud computing allow data management in real time, and transfer of data among different networks. Further, the App does not need to be connected to the internet while in use. A glossary of terminology used in the technical characteristics of the PainChek™ is presented in Table 1.

**Figure 1:**
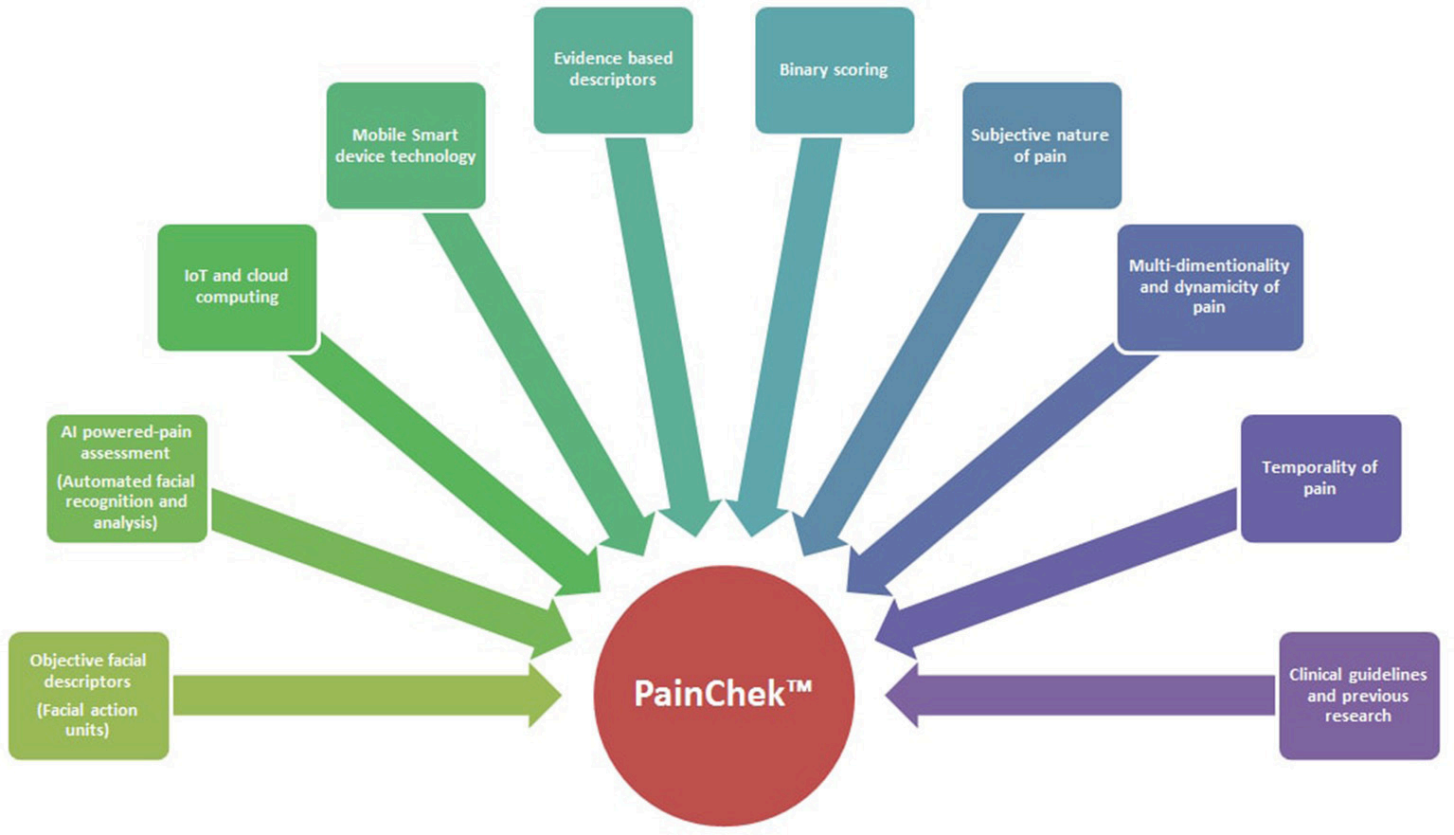
Conceptual model of the PainChek™ system.

**Table 1:** Glossary of technical terms used.

**Concept**	**Definition**
Cognification	The process of making objects smarter by combining, connecting and/or integrating 2 or more technologies; one of which is AI (Kelly, 2016). Also known as “artificial smartness”.
Artificial Intelligence (AI)	“The scientific understanding of the mechanisms underlying thought and intelligent behavior and their embodiment in machines” (AAAI, 1995-2013).
Smart computing	A generation of integrated hardware, software, and network technologies that provide IT systems with real-time awareness of the real world and advanced analytics to help people make more intelligent decisions about alternatives and actions that will optimize processes (Bartels et al., 2009).
Cloud computing	A model for enabling ubiquitous, convenient, on-demand network access to a shared pool of configurable computing resources (e.g., networks, servers, storage, applications, and services) that can be rapidly provisioned and released with minimal management effort or service provider interaction (Mell and Grance, 2011).
Affective computing	Computing that relates to, arises from, or influences emotions (Picard, 1997).
Internet of Things (IoT)	The networked interconnection of everyday objects, which are often equipped with ubiquitous intelligence. Also known as “Internet of Objects” (Xia et al., 2012).
Deep learning	A pattern recognition technique that allows computational models that are composed of multiple processing layers to learn representations of data with multiple levels of abstraction (LeCun et al., 2015).
Smart device	An electronic or digital mobile device that has advanced computational processing power, possess multiple capabilities (e.g., voice and video communication, data storage), can operate independently and interactively by being linked to other devices or networks via various wireless connections e.g., Wi-Fi, Bluetooth (Bamidis et al., 2015).
Android	A mobile operating system designed primarily for touchscreen devices such as smartphone and tablet computers, and for other electronics such as smart televisions (Android TV), and smart watches (Bamidis et al., 2015).
iOS	A mobile operating system developed by Apple, which works in a similar way to the Android.

### Rationale for painchek™ development

There are various perspectives as to why we have an urgent need to develop a valid, reliable, and more objective pain assessment system for people with dementia. From the patient's perspective, dementia limits their verbal and cognitive abilities to report the presence, nature, location, and/or intensity of pain. These impairments combined with subjective assessment of pain by health professionals and carers are primarily responsible for the failure to identify pain in this group. This then leads to adverse health outcomes, such as behavioral disturbances, use of inappropriate medications, poor quality of life, and premature death (Schneider et al., 2005; Holmes et al., 2008). From the carer's perspective, care is less burdensome when pain related-behavioral problems are well managed. There are also many potential benefits from proper pain management to the organization, which include staff efficiency and productivity, staff retention, and better quality of care for their patients.

### The painchek™ system

The PainChek™ system is a software system which is comprised of the following components:

- Mobile Application (App)
- Web Admin Portal (WAP)

PainChek™ is intended to be used to assess and monitor pain in people who cannot verbalize such as people with dementia or communication difficulties (ARTG, 2017).

A glossary of terminology used to describe the psychometric and clinimetric properties of pain assessment tools is displayed in Table 2. A comprehensive account of the clinical and technical characteristics of the PainChek™ system is described in Table 3.

**Table 2:** Glossary of terms used to describe psychometric and clinimetric properties of pain assessment tools.

**Concept**	**Definition**
Validity	The degree to which an instrument measures what it is intended to measure (Polit and Hungler, 1991).
Concurrent validity	The degree to which scores on an instrument are correlated with some external criterion, measured at the same time (Polit and Hungler, 1991).
Discriminant validity	An approach to construct validation that involves assessing the degree to which a single method of measuring two constructs yields different results (i.e., discriminates the two; Polit and Hungler, 1991).
Predictive validity	The degree to which an instrument can predict some criterion observed at a future time (Polit and Hungler, 1991).
Reliability	The degree of consistency or dependability (i.e., repeatability) with which an instrument measures the attribute it is designed to measure.
Interrater reliability	The degree to which two raters or observers, operating independently, assign the same ratings or values for an attribute being measured (Polit and Hungler, 1991).
Test-retest reliability	A procedure used to determine the stability of measurements over time (Waltz et al., 1991).
Internal consistency	The degree to which two or more measures are essentially measuring the same construct (Portney and Mary, 2009).
Sensitivity (SE)	Probability that a test result will be positive when the disease is present (true positive rate; Altman et al., 2000).
Specificity (SP)	Probability that a test result will be negative when the disease is not present (true negative rate; Altman et al., 2000).
Accuracy	Overall probability that a patient will be correctly classified (Altman et al., 2000).
Clinical utility	The usefulness of the measure for decision making (van Herk et al., 2007).
Clinical Utility Index (CUI)	The overall value of a test for combined screening and case finding (Mitchell, 2010).

**Table 3:** Clinical and technical characteristics of PainChek™ system.

**COMPONENTS OF THE PAINCHEK**™ **SYSTEM: SOFTWARE**	
Smart device enabled application	A point of care mobile application, which consists of: •Pain scale (**Figures 2A–F**) •Pain assessment log (**Figure 2J**) •Pain chart (**Figure 2K**) •Local patient database •Medications and therapies log •Comments section (**Figure 2M**).
Web administration portal (**Figure 3**)	A secure website that allows the management of patients and users data
**COMPONENTS OF THE PAINCHEK™ SYSTEM: HARDWARE**	
Smart device	A smart phone or tablet to deliver point of care pain assessments, and to capture temporal patterns of pain scores
PC or smart device	A computing device for WAP access
**ADMINISTRATION & OPERATION OF THE PAINCHEK™ APP**	
Operation system of the App	Android or iOS
Operation procedure	Download and Install the App Log in and set up user profile Enter details for a new patient or select an existing patient.
Time to set up the App	The iOS PainChek App is a 55 MB download. Assuming a download speed of 40 Mbs the average speed of a mobile connection in Australia as of mid-2017 (as reported by http://www.speedtest.net/), the PainChek App should take around 10 to 15 s to download.
Administration skills	Familiarity with the use of smart device Familiarity with the patient undergoing assessment Basic knowledge of pain behaviors in dementia.
Target users	Clinicians and carers
Training needs	User competence on the use of smart device technology and operation of the App Clinical competence on tool's contents, domains, and descriptors.
Training resources	Video tutorials accessible through the PainChek website FAQs (text and illustrating pictures) accessible through the website Face-to-face workshop for enterprise users.
	All materials are currently available in English but other languages are planned.
**CONTENT OF THE PAINCHEK™ APP**	
Pain scale (**Figures 2A–F**)	42 items distributed across 6 domains: The Face (9 items), The Voice (9 items), The Movement (7 items), The Behavior (7 items), The Activity (4 items), The Body (6 items)
Pain chart (**Figure 2K**)	A graphical representation of pain scores over a period of time
Pain assessment log (**Figure 2J**)	A list of pain assessments completed with their corresponding time and dates
Patient database	A local repository of patients' data including demographics
Medications and therapies log	A local repository of medications and therapies of each patient
**MODES OF THE FACE DOMAIN**	
Front camera mode	Automated facial analysis using the front camera of a smart device
Back camera mode	Automated facial analysis using the back camera of a smart device
Manual mode	Manually completed facial assessment (optional)
**SCORING OF THE PAINCHEK™ PAIN SCALE**	
Scoring format	Binary (yes/no) checklist
Scoring instructions	Observe the patient Use the AFRA in the Face domain to detect facial action unit descriptors Complete the corresponding checklists for the remaining non-facial domains The App automatically calculates a pain intensity score, which conforms to one of the pain category bands below.
Scoring interpretation (total pain scores)	0-6 (No Pain), 7-11 (Mild Pain), 12-15 (Moderate Pain), ≥ 16 (Severe Pain)
Ideal conditions of pain assessments	Assess pain at rest (e.g. sitting) and immediately after movement (e.g. repositioning) Assess and re-assess (e.g., 1 h post-intervention).
Time to complete scoring of total scale	≤ 1 min
Time to complete scoring of the Face domain (automated)	3 s
**CLINICAL STUDIES**	
Study 1 (Atee et al., 2017a)	Design: prospective observational study; Setting: RACFs; Sampling: purposive convenience; Time line: 13 weeks, N: 40; Age: 60–98 years
Study 2 (Atee et al., 2017b)	Design: prospective observational study; Setting: RACFs; Sampling: purposive convenience; Time line: 10 weeks; N: 34; Age: 68–93 years
Study 3 (Hoti et al., 2018)	Design: *post-hoc* statistical analyses based on Study 2 findings
**PSYCHOMETRICS OF THE PAINCHEK™ PAIN SCALE**	
Concurrent validity	Excellent Study 1: r = 0.882 (95% CI: 0.857-0.903) Study 2: r = 0.911 (95% CI: 0.893-0.927)
Discriminant validity	Good (regression model not significantly influenced by the timing of the assessment i.e. at rest vs. with movement) Study 1: *p* = 0.795 Study 2: *p* = 0.243
Internal consistency reliability	Excellent homogeneity Study 1: α = 0.925 Study 2: α = 0.950
Inter-rater reliability	Good-to-excellent *Study 1:* κ_w_ = 0.74 (95% CI: 0.69-0.80) *Study 2:* κ_w_ = 0.86 (95% CI: 0.82-0.90)
Test-retest reliability	Excellent *Study 2:* ICC = 0.904 (95% CI: 0.885-0.921)
**CLINIMETRICS OF THE PAINCHEK™ PAIN SCALE**	
Predictive validity	Good based on the following data: *Study 2* Preintervention pain scores (i.e. at rest) Mean = 8.33 ± 3.34; Median = 9; Mode = 10 Postintervention pain scores (i.e. post movement) Mean = 11.44 ± 3.54; Median = 11; Mode = 13 *t-*test: *p* < 0.0001
Clinical utility	Excellent based on the following data: *Study 1* Contingency Table Pain categories were derived using a contingency table with the APS. Pain intensity scores include 4 categories: no pain (0-6), mild pain (7-11), moderate pain (12-15), severe pain (≥16) *Study 3* Mitchell's Clinical Utility Index CUI (+) = 0.936 (95% CI: 0.911-0.960) CUI (−) = 0.801 (95% CI: 0.748-0.854) CUI = 0.95 ROC Curve AUC = 0.98 (95% CI: 0.96–0.99) Optimal cut-off for pain = 7
Accuracy	Excellent based on the following data: *Study 3* Accuracy = 95.0% (95% CI: 92.9%-97.1%) SE = 96.1% (95% CI: 93.9%-98.3%) SP = 91.4% (95% CI: 85.7%-97.1%)
**CHARACTERISTICS OF THE WAP**	
Browser compatibility	Chrome (version 59.0 or later), Mozilla (version 54.0 or later), Internet Explorer (version 11 or later)
Operating system	Windows (7 or later), or Macintosh (OS X Mavericks 10.9 or later)
Data hosting product	Amazon Elastic Compute Cloud (Amazon EC2) (AWS, 2017)

*N, number of subjects with moderate to severe dementia; RACFs, residential aged care facilities; r, Pearson's correlation coefficient; α, Cronbach alpha; κ_w_, Weighted Kappa; ICC, Intraclass correlation coefficient; CUI, Clinical Utility Index; AUC, Area Under Curve (of receiver-operator characteristic curve); ROC, receiver-operator characteristic curve; SE, Sensitivity; SP, Specificity*.

### The painchek™ app

PainChek™ is a point of care software application (App) that is compatible with Android and iOS smart devices. The tool uses automated facial recognition technology in real time to identify nine facial micro-expressions called action units (AUs), which are derived from the FACS (Atee et al., 2017a). These facial AUs are validated indicators of the presence of pain (Prkachin, 1992, 2009; Prkachin and Solomon, 2008; Craig et al., 2011; Kunz, 2014; Kunz and Lautenbacher, 2014). These data are then combined with other non-facial pain cues (also known as communicative and protective pain behaviors) such as vocalizations, movements, and behaviors inputted by the user to automatically calculate a pain severity score (Atee et al., 2017a). The App includes a number of components, which are outlined in Table 3.

The PainChek™ pain scale is composed of 42 items distributed across six domains (Table 3; Atee et al., 2017a). Using the smart-device camera to capture a short video of a person's face, the App automatically identifies the face in real time, then maps the face to analyze facial expressions (using a built-in AI algorithm) indicative of the presence of pain. This step provides a score for Domain 1. The user of the App then completes the checklists in Domains 2–6, to give rise a numerical pain score which fits one of the following categories: no pain (0–6), mild pain, (7–11), moderate pain (12–15), or severe pain (≥16) (Atee et al., 2017a).

**Figure 2:**
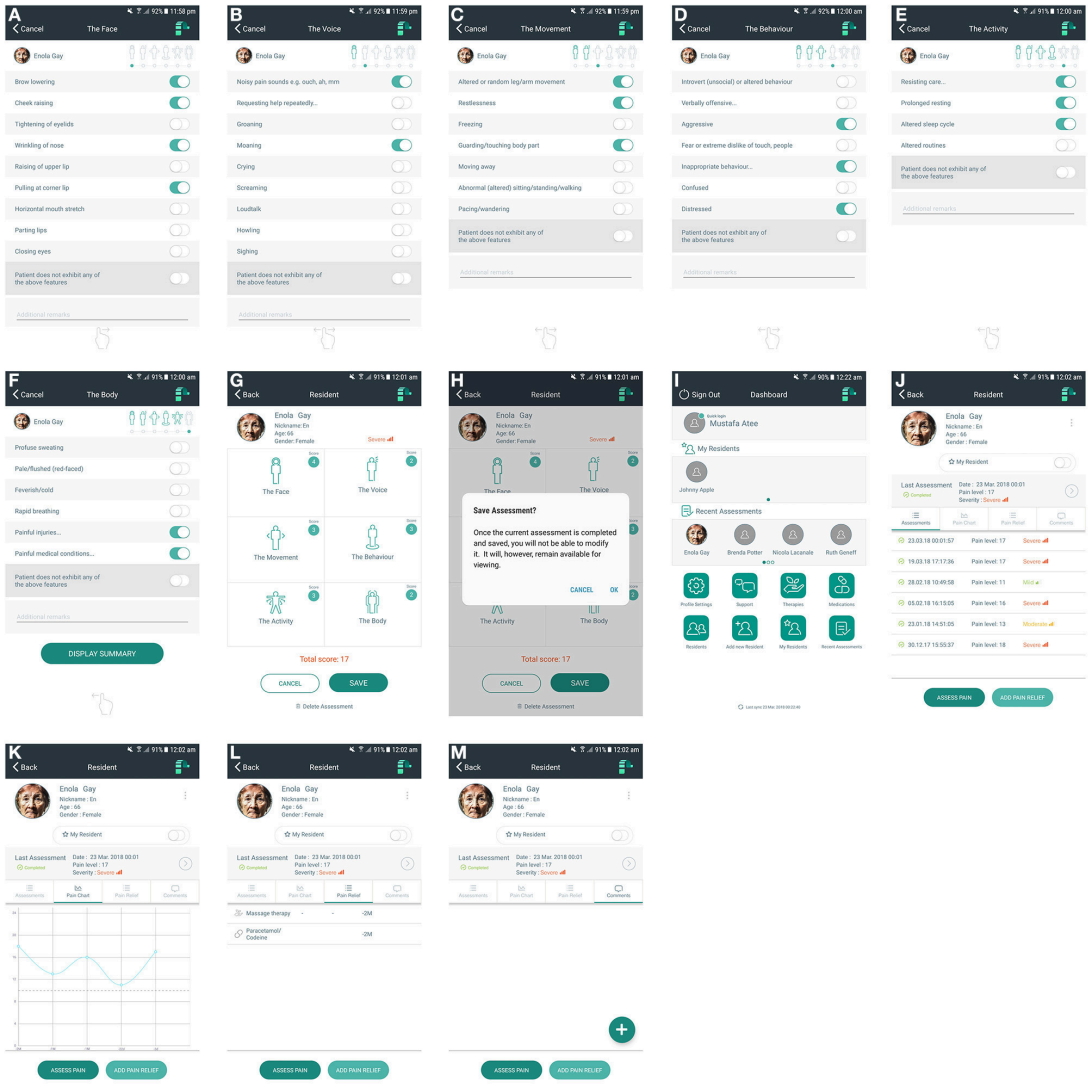
**(A)** PainChek™ pain assessment tool-The Face (Domain 1). **(B)** PainChek™ pain assessment tool-The Voice (Domain 2). **(C)** PainChek™ pain assessment tool-The Movement (Domain 3). **(D)** PainChek™ pain assessment tool-The Behavior (Domain 4). **(E)** PainChek™ pain assessment tool-The Activity (Domain 5). **(F)** PainChek™ pain assessment tool-The Body (Domain 6). **(G)** PainChek™ pain assessment tool-Summary screen. **(H)** PainChek™ pain assessment tool–Saving assessment. **(I)** PainChek™ App—“Dashboard” screen. **(J)** PainChek™ App—“Assessments” log. **(K)** PainChek™ App—“Pain Chart.” **(L)** PainChek™ App—“Pain Relief” list. **(M)** PainChek™ App—“Comments” section.

The PainChek™ App (Figures 2A–M) is commercially available through PainChek Ltd, and is demonstrated in this animated video: https://www.youtube.com/watch?v=rGhX91ZEX9w.

### The web administration portal (WAP)

**Figure 3:**
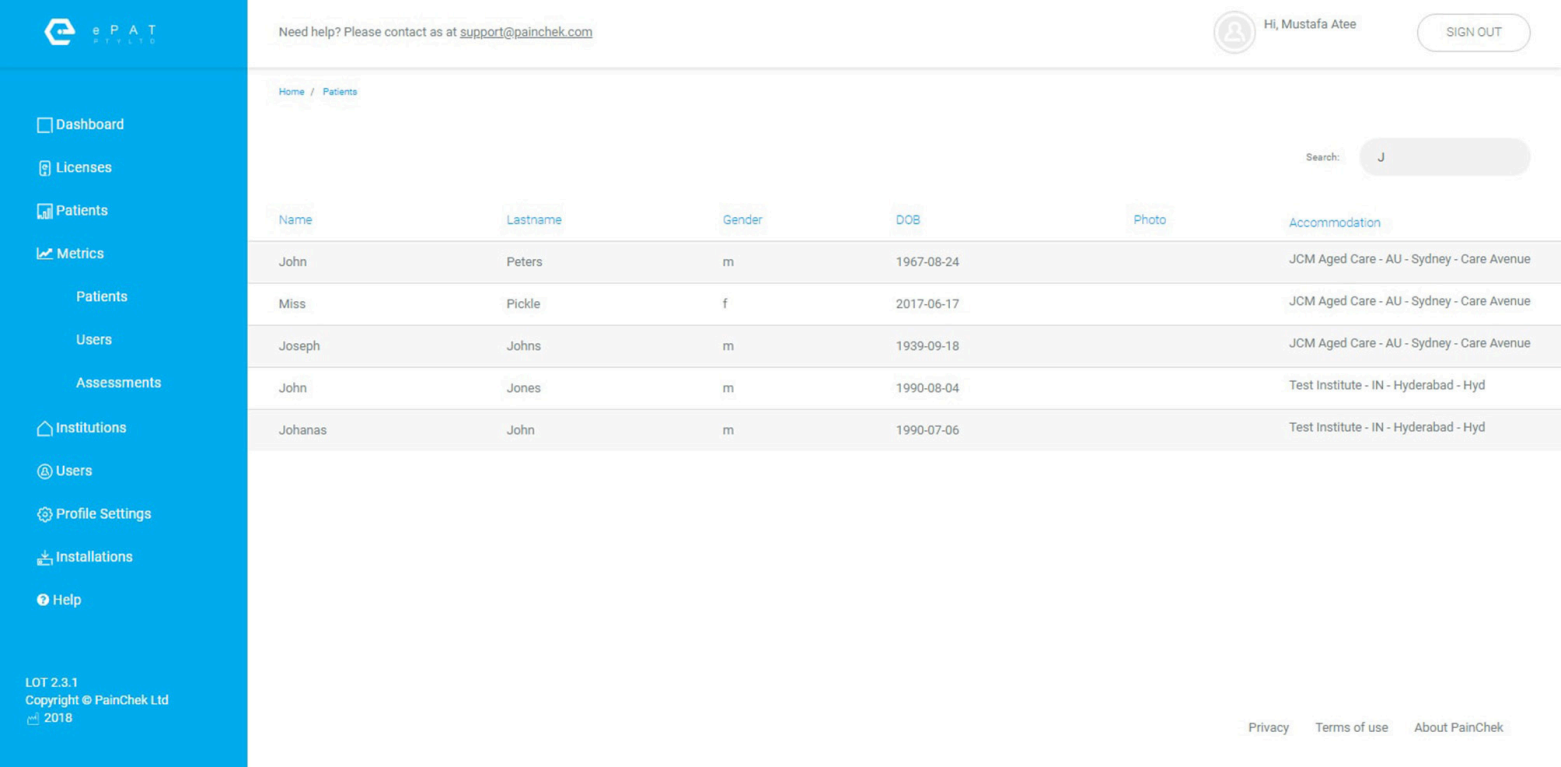
PainChek™ Web Admin Portal (WAP).

The PainChek™ Web Administration Portal is a secure website that allows administrators to manage patient data, and activate new users. The WAP is a cloud hosted web application that can be accessed via a dedicated URL using any of the following internet browsers: Chrome (version 59.0 or later), Mozilla (version 54.0 or later), Internet Explorer (version 11 or later). The WAP of PainChek™ is supported through the operating systems of Windows (7 or later), or Macintosh (OS X Mavericks 10.9 or later). The portal is currently hosted on Amazon Webs Services using the Amazon Elastic Compute Cloud (Amazon EC2) (AWS, 2017). Figure 3 illustrates a screenshot of the WAP. Characteristics of the WAP are also summarized in Table 3.

### Clinical studies

To date, three studies about the PainChek™ App have been published (Table 3; Atee et al., 2017a,b; Hoti et al., 2018). In blind comparisons with the Abbey Pain Scale, PainChek™ has been clinically evaluated in aged care residents with moderate to severe dementia in two prospective observational studies (Atee et al., 2017a,b). The third study provided a comprehensive clinimetric analysis on the performance of the App (Hoti et al., 2018).

*Study 1: Pain Assessment in Dementia: Evaluation of a Point-of-Care Technological Solution* (Atee et al., 2017a).

In this study, Atee el al. provided an account of the description, content, and conceptual synthesis as well as the psychometric properties of the PainChek™. The App was tested in 40 residents, who underwent 353 paired assessments during rest (*n* = 209) and movement (*n* = 144). PainChek™ was demonstrated to have excellent concurrent validity and internal consistency, together with good interrater reliability and discriminant validity (Table 3; Atee et al., 2017a).

*Study 2: Psychometric evaluation of the Electronic Pain Assessment Tool (ePAT): An Innovative Instrument for Individuals With Moderate-to-Severe Dementia (Atee et al.*, *2017b**)*.

Based on 400 paired assessments, the psychometric properties of the tool were further examined in 34 geriatric residents. Again, the App demonstrated strong psychometric properties: excellent concurrent validity, interrater reliability, internal consistency, and excellent test-retest reliability. Discriminant validity and predictive validity were both good (Table 3) (Atee et al., 2017b).

*Study 3: Clinimetric Properties of the Electronic Pain Assessment Tool (ePAT) for Aged-Care Residents With Moderate to Severe Dementia (Hoti et al.*, *2018**)*.

Hoti et al. further analyzed Study 2 data to confirm the cut-off scores, and predictive validity of the tool, whilst also reporting on its clinical utility. Using the ROC curve methodology, the cut-off points for presence of pain was confirmed to be ≥ 7. The study demonstrated the high accuracy, sensitivity and specificity of the App in detecting pain in individuals with dementia. It also demonstrated the excellent clinical utility of the App for pain screening and case finding, as indicated by Mitchell's Index (Table 3) (Hoti et al., 2018).

### Clinical guide and training on the use of painchek™

A wide variety of training resources have been developed to assist users with the operation of PainChek™. Resources include face-to-face training, and web-based materials.

**Figure 4:**
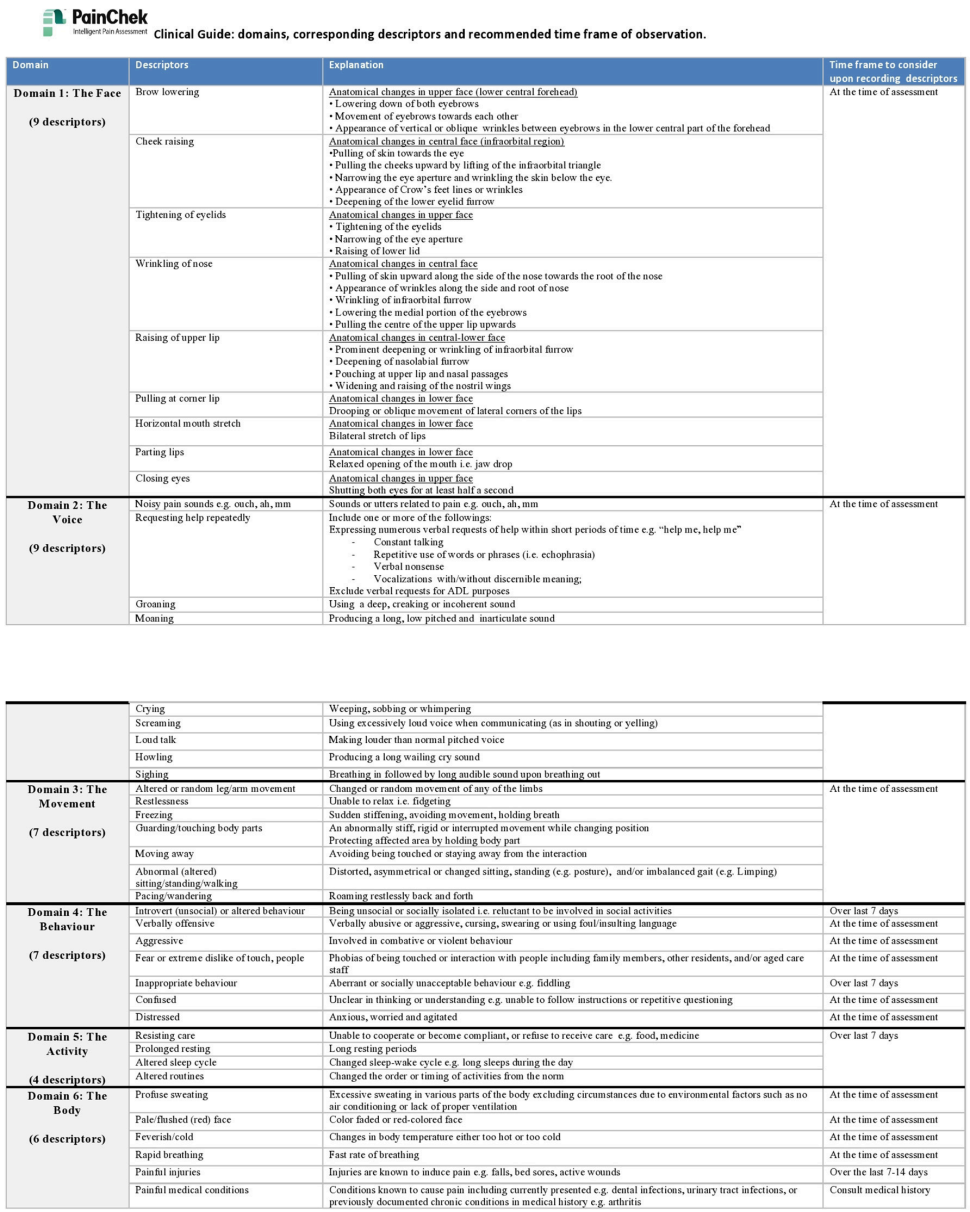
“Clinical Guide” handout.

The face-to-face training is available for institutional users. The training is a 2.5 h program which comprises of four sessions: (1) pain associated behaviors in people with dementia, (2) pain assessment in people with dementia, (3) PainChek™ (e.g., contents, scoring, and administration), and (4) practical training and clinical shadowing. The program consists of video and written materials, which have been prepared or collated by clinical researchers (who developed the PainChek™ system, and experienced in the area of pain and dementia). The video clips cover a wide range of pain relevant materials including AUs typically displayed during pain (simulated). Similarly, a number of facial images of people in pain and clinical cases are also used during the training. A two-page clinical guide handout (Figure 4), explaining the content, domains, corresponding descriptors and recommended time frame of observation is also available to the trained users. A clinical facilitator experienced on the use of the system is responsible for running the training program.

For each patient, it is essential that pain assessments are carried out during rest first. This is in order to determine baseline scores for benchmarking purposes, which then followed by post movement assessment to capture the nociceptive experience. Kinesthetic activities such as bending and walking are part of daily living. These activities involve joint activity, which generates nociceptive signals, encoding the sensory aspect of pain phenomenon (Breivik et al., 2008).

The web-based resources (https://www.painchek.com/) include user guides, video materials, and frequently asked questions. All training resources are currently available in English language, although translation to other languages is planned.

## Discussion

This article reports on an innovative pain assessment system that includes a point of care App and WAP. The PainChek™ App (Figure 2) is a newly TGA-approved and CE marked medical device for pain assessment in non-verbal adult populations (ARTG, 2017). The App is linked to WAP (Figure 3) to allow capturing of data collected in the clinical setting.

The conceptual model around which the PainChek™ was developed is multifaceted (Figure 1). This model entails subjectivity, multi-dimensionality, and dynamicity of pain as a construct, objectivity, and comprehensiveness of pain behaviors included in the tool, simple scoring, and administration procedure of the pain scale, as well as technological advancements and innovative characteristics of the platform used. Of particular importance, AFRA were used for the first time in a smart device enabled tool for pain assessment targeted at people with dementia. This novel design (including the AI-assisted scoring) facilitates the process of pain assessment and allows pain scores to be obtained in a less subjective way. The system has password-enabled authentication to protect and secure the data. Further, the binary scoring of pain scale reduces the likelihood of user judgement bias when recording non-facial items (Ridley, 2002). It also allows easier quantification of patient's characteristics in terms of pain which in turn facilitates the prognostication of pain severity. This allows for conversion of probability (present or absent) into a binary outcome which informs the tool's ordinal scale of pain severity (mild, moderate, severe; Ridley, 2002). The latter is arrived through automated summation of the total pain score (Figure 2G). Because PainChek™ is a digital tool linked to the WAP, the technology overcomes the limitations of guessing practices and poor identification of the presence of pain by nurses and geriatricians in people with severe cognitive impairment (Kovach et al., 2000; Cohen-Mansfield and Lipson, 2002). Through systemizing and structuring the process of assessment, PainChek™ will overcome these challenges and improve multidisciplinary communication among health care professionals. For its use in clinical practice, the App does not require Internet connection. Synchronizing the clinical data (collected through the App) with the WAP even at a later time still preserves the actual time of pain assessment as the App saves each assessment in real time (Figures 2H,J). Electronic documentation of the system has the capacity to allow patient profiling using their corresponding pain assessment (Figure 2J) and management logs (Figure 2L). The ability of the system to collect a large amount of pain data over a period of time helps in identifying temporal patterns (Figure 2K), which can offer useful clinical insights in terms of patients' responsiveness to interventions provided during a specified period. PainChek™ also facilitates the interdisciplinary communication among health care practitioners through providing a more comprehensive and up-to-date picture of patient's pain. This approach was also suggested by Lichtner et al. for decision support systems for pain management in patients with dementia (Lichtner et al., 2015, 2016).

A primary deficiency in the development of majority of the health related apps is the fact that they are designed by non-health care practitioners (Lalloo et al., 2015). In contrast, our system was developed by clinical researchers in collaboration with AI and web interface engineers (Atee et al., 2017a). In addition, most existing health apps including those related to pain, lack evidence in their development and testing (Rosser and Eccleston, 2011), whereas the PainChek™ App has been conceptualized, and clinically tested around the best available evidence in gerontology, pain, and dementia (Atee et al., 2017a,b). Further, the PainChek™ App is to our knowledge the only pain assessment tool in dementia that has regulatory clearance in Australia (TGA) and Europe (CE mark), as a medical device (ARTG, 2017). Through clinical studies, we have also demonstrated that our approach in developing a hybrid model (automated FACS pain relevant items and other clinical indicators related to older adults with dementia) is a valid and reliable method in evaluating pain (Atee et al., 2017a,b).

Our conceptualized model design was also supported by the literature (Herr and Garand, 2001; AGS Panel on Persistent Pain in Older Persons, 2002; Herr, 2002; Herr et al., 2006, 2011; Hadjistavropoulos et al., 2007, 2014; Beach et al., 2016). Lints-Martindale et al. (2012) found that the AGS-recommended pain behavioral domains are comprehensive and useful indicators in recognizing painful episodes. Beach et al. (2016) reported that integrating objective behavioral descriptors into observational tools improve pain assessment in people with Alzheimer's dementia (AD). Defined as “Actions or postural displays that are enacted during the experience of pain,” pain behaviors are important manifestations that convey rich information about patient's pain severity (Hadjistavropoulos and Craig, 2002; Herr et al., 2017). Of these behaviors, non-verbal expressions of pain such as facial expressions, vocalizations, and body movements are generally difficult to suppress (Martel et al., 2012). Of note, facial expressions of pain have been widely researched because they are “readily accessible, highly plastic, and are believed to be the most specific, encodable form of pain behavior” (Williams, 2002). Facial expressions are also one of the strongest indicators of pain particularly in people with cognitive impairment or dementia (Kunz et al., 2007, 2009). There is a significant increase of pain behaviors in AD compared to healthy control (Lautenbacher et al., 2013; Beach et al., 2017). Horgas et al. (2009) indicated that the resultant numerical scores from summating pain behaviors are closely linked to the self-report of pain. In their meta-analysis Labus et al. (2003), also found that the use of multiple behavioral domains has a synergistic effect on pain assessment because the obtained scores are more representative of subjective pain experience. McCahon et al. (2005) noted that observation of pain behaviors is a valid and reliable assessment method for use with patients with chronic pain. Although these findings were drawn from samples of cognitively intact individuals with chronic pain, similar trends were also observed in people with cognitive impairments. A recent review of pain behaviors in people with dementia by Herr et al. (2011, 2017) revealed that these behaviors are strong indicators of the presence and intensity of pain. Further, these pain behaviors configure the item descriptors list of observational pain assessment tools in verbal and non-verbal geriatric populations (Herr et al., 2011, 2017; Lichtner et al., 2014). In older adults with dementia, pain behavior tools improved binary pain recognition (i.e., presence or absence of pain) by up to 25.4%, and ordinal level of pain intensity by up to 42.5% above chance (Lukas et al., 2013). It is thus evident from the above that there is a consensus in the literature about the predictability of pain related behaviors in informing the assessment of clinical pain.

The App has been designed for administration by a wide range of users including clinicians and carers (Atee et al., 2017a). The training resources are comprehensive and diversified. The tool is available as a smart device App compatible with various mobile operating systems such as Android and iOS (Atee et al., 2017a,b). Thus, these useful and unique characteristics cover multiple aspects of clinical utility, such as scoring, administration time and skills, and supporting materials which most of the other tools are currently missing (Lichtner et al., 2014; Herr et al., 2017).

In conclusion, evidence to date suggests that the PainChek™ system offers a novel method that should make the process of pain assessment and monitoring simpler and more objective for clinicians and carers of patients who cannot verbalize their pain.

## Availability of materials

The PainChek™ system and materials reported in this manuscript are commercially available from PainChek Ltd, Sydney, NSW, Australia.

## Data availability statement

All relevant data is contained within the manuscript. No additional datasets were generated for this manuscript because the submitted work is a technology report.

## Ethics statement

All clinical studies of PainChek™ that involved human subjects reported in this manuscript were conducted in accordance with the World Medical Association's Declaration of Helsinki, the recommendations and policy statements of the Alzheimer's Association and the World Health Organization on assistive technologies for people with dementia. These studies were approved by the Human Research Ethics Committee of Curtin University, Western Australia (HREC: HR10/2014), and the participating aged care groups. Study 2 was also registered with Therapeutic Goods Administration (TGA) under the Clinical Trial Notification (CTN) Scheme (CT-2016-CTN-04886-1 v1). Due to cognitive impairment of the involved subjects, proxies gave authorized written informed consent on their behalf. All data were de-identified to protect confidentiality.

## Author contributions

MA, KH, and JH: conceived the idea; MA: drafted and organized the manuscript, and conducted the literature search. All authors reviewed, contributed to and approved the final version of the manuscript.

### Conflict of interest statement

All authors are shareholders in PainChek Ltd, which is marketing the PainChek™ instrument. They also have a patent application titled “A pain assessment method and system” (PCT/AU2015/000501), which is currently under national phase examination since February 2, 2017. MA is a Research Scientist for PainChek Ltd while serving as a Research Fellow and Ph.D. Candidate with the School of Pharmacy and Biomedical Sciences, Curtin University. KH is employed as a consultant by PainChek Ltd while serving as an Assistant Professor at University of Pristina, and an Adjunct Senior Lecturer at the School of Pharmacy and Biomedical Sciences, Curtin University. JH holds the position of Chief Scientific Officer of PainChek Ltd while serving as a Professor at the School of Pharmacy and Biomedical Sciences, Curtin University.
